# MGmapper: Reference based mapping and taxonomy annotation of metagenomics sequence reads

**DOI:** 10.1371/journal.pone.0176469

**Published:** 2017-05-03

**Authors:** Thomas Nordahl Petersen, Oksana Lukjancenko, Martin Christen Frølund Thomsen, Maria Maddalena Sperotto, Ole Lund, Frank Møller Aarestrup, Thomas Sicheritz-Pontén

**Affiliations:** 1 Department of Bio and Health Informatics, Technical University of Denmark, Kongens Lyngby, Denmark; 2 National Food Institute, Technical University of Denmark, Kongens Lyngby, Denmark; University of Arizona, UNITED STATES

## Abstract

An increasing amount of species and gene identification studies rely on the use of next generation sequence analysis of either single isolate or metagenomics samples. Several methods are available to perform taxonomic annotations and a previous metagenomics benchmark study has shown that a vast number of false positive species annotations are a problem unless thresholds or post-processing are applied to differentiate between correct and false annotations. MGmapper is a package to process raw next generation sequence data and perform reference based sequence assignment, followed by a post-processing analysis to produce reliable taxonomy annotation at species and strain level resolution. An in-vitro bacterial mock community sample comprised of 8 genuses, 11 species and 12 strains was previously used to benchmark metagenomics classification methods. After applying a post-processing filter, we obtained 100% correct taxonomy assignments at species and genus level. A sensitivity and precision at 75% was obtained for strain level annotations. A comparison between MGmapper and Kraken at species level, shows MGmapper assigns taxonomy at species level using 84.8% of the sequence reads, compared to 70.5% for Kraken and both methods identified all species with no false positives. Extensive read count statistics are provided in plain text and excel sheets for both rejected and accepted taxonomy annotations. The use of custom databases is possible for the command-line version of MGmapper, and the complete pipeline is freely available as a bitbucked package (https://bitbucket.org/genomicepidemiology/mgmapper). A web-version (https://cge.cbs.dtu.dk/services/MGmapper) provides the basic functionality for analysis of small fastq datasets.

## Introduction

The advances in rapid and efficient DNA sequencing technologies have made it possible to study microbial communities from a wide variety of environments, such as sediments [1][2], water [3], ice [4], and humans [5][6]. Among the known DNA sequencing platforms, Illumina HiSeq and MiSeq are often preferred for both single genome and metagenomics studies, due to the large data output and a relatively low cost per base pair. Applying the whole genome shotgun sequencing technique, all DNA in a biological sample is sequenced and several millions of short read nucleotide sequences are produced. Metagenomics data from a single human gut sample is a complex system representing hundreds of organism and even more diversity is expected when samples originate as a mixture from many individuals e.g. humans or animals from sewage systems, public transportation sites or animal farms. The interest in analyzing such datasets may be monitoring of bacterial or viral pathogens, identification of anti-microbial resistance genes, phage identification or simply obtaining a complete catalogue of the organisms that are present. Such analysis is not straight-forward, it requires programs that can perform the mapping of fastq sequence reads to many reference sequence databases without an extensive memory usage, parsing and validating sequence alignment hits, taxonomy annotation with a reduced false positive rate and finally presenting output of the taxonomy analysis and also providing files for further downstream analysis (SNP or contig assembly. MGmapper was made to provide an access for routine analysis of complex datasets, enabling usage of many whole genome reference sequence databases like bacteria, virus, fungi, plant, vertebrate-mammals, invertebrates and also enables the usage of gene databases like anti-microbial resistance genes, 16S rRNA or any custom database based on a set of fasta sequences. The huge fasta sequence databases like plant (208gb fasta) vertebrate mammals (316gb fasta) or invertebrates (150gb fasta) can be split into smaller chunks (10gb fasta) thus the total memory requirement is reduced to 30–40gb memory when running MGmapper. Also, most tasks are run in parallel for fast execution.

The task to assign each of those nucleotide reads to the genome that they represent is challenging and the problem of false positive predictions is always an issue to be considered for alignment based methods where a query sequence is mapped against a large database of target sequences. As target databases increase in size, the chance of finding hits for random reasons also increases. For decades the Blast program suite [7] has been one of the most frequently used programs for pairwise alignment of a query sequence against a large database of target sequences. Blast utilizes a filter in form of an expect value as a threshold, to reduce the number of false positives. In general methods within the field of taxonomy annotation rarely use filters or cutoffs, but a recent benchmark study showed the need, as several methods vastly over predict the number of species present when evaluated on both *in vitro* and *in silico* datasets [8]. The study involved benchmarking of 15 taxonomy annotation methods where two of them, a filtered version Kraken [9] and CARMA3 [10], correctly identified all species present in an *in vitro* dataset, using a read count abundance threshold at 0.1%. Also, the methods MEGAN4 [11][12] RAPSearch2 [13] performed well with only one false positive species annotation.

The read count abundance measure is biased as more reads are sequenced from larger genomes compared to smaller genomic sequences like viruses and plasmids. Thus a normalization of read count abundances with the reference sequence size can adjust for a skewed performance that favors large reference sequences. In the MGmapper method we have introduced such a measure to reduce the false positive taxonomy annotations. During whole genome shotgun sequencing, all DNA is fragmented and sequenced from one or both ends depending on whether single-end or paired-end reads are produced. Thus our size normalized read count abundance (S_Abundance) is divided by 2 in case paired-end reads are used and multiplied by 100 for convenience i.e. 100*ReadCount/Size(bp)*2, where *Size* is the length of a reference sequence. Using the normal read count abundance (ReadCount/Reads_in_sample) as in the work by Peabody et al. [8], a threshold at 0.1% appeared to be the best cut-off to differentiate between true and false taxonomy annotations. For the S_Abundance, a threshold of 0.01 was the best cut-off, based on benchmarking data as used by Peabody et al. One drawback of using a size normalized abundance as criterion for true positive annotations is that, in case of small reference sequences, only a few assigned reads are needed to pass the cutoff. Therefore a lower read-count abundance-threshold could be introduced, although the exact value may be difficult to assign.

Identifying a specific reference sequence e.g. a bacterial strain or an anti-microbial resistance gene in a pool of highly similar sequences is a challenge for any taxonomy annotation method. Only a few sequence reads aligned to specific marker regions may enable the differentiation between closely related genes or strains. For this reason the presence of uniquely mapped sequence reads to one specific target reference sequence is a strong indicator that the target sequence is actually present in the sample. K-mer based methods like Kraken [9] or KmerFinder [14] require a 100% identity between a query fragment and a database hit with the annotation for that fragment. Typically k-mers with a of length of 31bp are used and at that size a kmer may be assigned to several reference sequences. For the Kmerfinder [14] method an expectation value is calculated as the number kmers assigned to a specific reference sequence within a database compared to the number of hits to other reference sequences in the same database. The Kraken [9] method utilizes another approach based on identifying the Lowest Common Ancestor (LCA) for each of the kmers originating from a sequence read. A score is calculated as the fraction of kmers that are rooted to a specific taxa compared to total number of LCA kmers that are assigned to a fastq read. As Kraken can calculate a score for a fastq read based on k-mer counts, alignment based methods utilize a sequence alignment score for the individual reads. Alignment based methods can handle nucleotide variations between a query read and a reference sequence, and the alignment score is typically used to differentiate the best alignment from secondary alignments with a lower score. The scoring scheme itself is based on heuristics arguments as in Blast [7], and BWA-mem [15], where scores for a nucleotide match, mis-match and inserts/deletions (INDELs) are summed to an overall alignment score. Thus alignment-based methods provide both numbers for uniquely mapped reads and total number of nucleotide matches, mis-matches and INDELs. Also, in BWA-mem [15] the edit-distance is the number of changes that are needed to obtain a perfect match between a sequence read and a reference sequence.

In summary, the normalized read count abundance, a low-read-count value, the number uniquely mapped reads and the edit-distance are measures used by MGmapper, rather than a single read count abundance threshold, with the aim to reduce the number of false positive taxonomy annotations from next generation sequence data.

## Materials

### Mock bacterial datasets

Two mock bacterial datasets were previously used to benchmark metagenomics classification methods at genus and species level [8]. An *in vitro* dataset comprised of single-end reads with an average length of 223bp can be downloaded from the metagenomics RAST server [16] with accession id 4545485.3. Also, 4 paired-end *in silico* datasets were downloaded: 4545483.3 (100bp), 4548385.3 (250bp), 4548991.3 (500bp) and 4548990.3 (100bp). There are a few changes in species nomenclature and dataset composition compared to the taxonomy that was presented in the original work by Peabody et al.[8]. For the sake of clarity the updated taxonomy annotations for both the *in vitro* and *in silico* datasets are given in Table 1.

**Table 1:** Mock bacterial composition of *in vitro* and *in silico* datasets.

Genus	Species	Strain	*In vitro*	*In silico*
Bacillus	*B*.*amyloliquefaciens*	DSM7	x	x
Bacillus	*B*.*cereus*	ATCC 14579	x	x
Burkholderia	*B*.*cenocepacia*	J2315	x	x
Escherichia	*E*.*coli*	K-12	x	x
Frankia	*Frankia*.*sp*.	CcI3	x	x
Micrococcus	*M*.*luteus*	NCTC 2665	x	x
Pseudomonas	*P*.*aeruginosa*	PAO1	x	x
Pseudomonas	*P*.*aeruginosa*	UCBPP-PA14	x	x
Pseudomonas	*P*.*Fluorescens*	Pf-5	x	x
Pseudomonas	*P*.*putida*	KT2440	x	x
Rhodobacter	*R*.*capsulatus*	SB 1003	x	x
Streptomyces	*S*.*coelicolor*	A3(2)	x	x
Nocardioides	*Nocardioides sp*.	JS614		x

Taxonomy clades are as follows: 12 strains, 11 species and 8 genuses for *in vitro* data and 13 strains, 12 species and 9 genuses for the *in silico* dataset.

Both the *in vitro* and the four *in silico* datasets were mapped against a bacteria and a plasmid reference sequence database. Databases were compiled as subset of entries from the assembly_summary file available at ftp://ftp.ncbi.nlm.nih.gov/genomes/genbank/bacteria/assembly_summary.txt. Criteria for sequence selection were defined by these parameters: version_status = ‘latest’, genome_rep = ‘Full’ and assembly_level = 'Complete Genome' or assembly_level = 'Chromosome'. In total the bacteria database is composed of 7451 genomic sequences (created: Feb 23, 2016), where entries with the word ‘plasmid’ in the fasta header were compiled into a separate plasmid database composed of 4429 genomic sequences.

## Methods

The MGmapper package consists of a pipeline of scripts to process FASTQ files as either single or paired-end reads to perform sequence mapping and taxonomy annotation against user defined reference sequence databases. MGmapper utilizes a number of publicly available programs: Cutadapt [17] for trimming and adaptor removal, BWA-mem [15] and SAMtools [18] to produce and process the reference based sequence alignments to one of many reference sequence databases. A short summary of the procedure is described below for paired-end sequence data, followed by more details outlined in the section “*Fastq mapping procedure”*.

Initially, a filtering step checks for properly paired reads, followed by trimming and adaptor removal. The biological relevant reads are obtained by always mapping to a PhiX bacteria phage and continuing with the subset of reads that do not align to the PhiX genome (commonly used as a control in Illumina sequencing runs). Next, sequence reads are mapped to user defined reference sequences and only properly paired reads are accepted, provided that both reads pass a lower alignment score threshold and relative alignment length. After mapping reads to all reference sequence databases (eg human, bacteria, fungi etc.), some reads may align to reference sequences in different databases and depending on the mapping mode (*bestmode* or *fullmode* explained further down) the best hit is identified and used to assign taxonomy. Taxonomy annotations (ftp://ftp.ncbi.nih.gov/pub/taxonomy/taxdump.tar.gz) are added via lookup in a pre-made Kyoto Cabinet database (http://fallabs.com/kyotocabinet/) containing *key*, *value* pairs in form of the reference sequence name (the *key*) and full taxonomy path from strain to superfamily clades (the *value*). Finally, a post-processing step (section “*post-processing*”) identifies confident assignments at strain, species, genus or any user defined taxonomy clade up to superfamily.

MGmapper can map sequence reads against any nucleotide sequence database i.e. both genomic and gene sequence databases and for each database the mapping can be performed in either *bestmode* or *fullmode*. In *bestmode*, reads are assigned to only one reference sequence if it is the best hit that is observed when mapping to all databases specified for *bestmode* mapping. Best hit is identified based on the highest alignment score. In *fullmode* reads are assigned to a reference sequence even if a better hit is seen when mapping to another database. Typically the *fullmode* is used to search for sequences (e.g. a gene database), that may be a subset of another database (e.g. a full genome database). Analyzing a sample for both genomic bacterial composition and anti-microbial resistance genes is a situation where MGmapper should be run with the bacterial database specified for *bestmode* mapping and at the same time specifying the anti-microbial resistance gene database for *fullmode* mapping. The reason is that the resistance genes are or may be a subset of the bacterial genomic sequences and we want to assign a sequence read both a bacteria genome and also a resistance gene. If both databases were specified for *bestmode* mapping (bacteria, anti-microbial genes), then a read can only be assigned to one of the databases and if identical alignment scores are observed, then priority is to the database that was specified first.

### Fastq read mapping procedure

**Fig 1:**
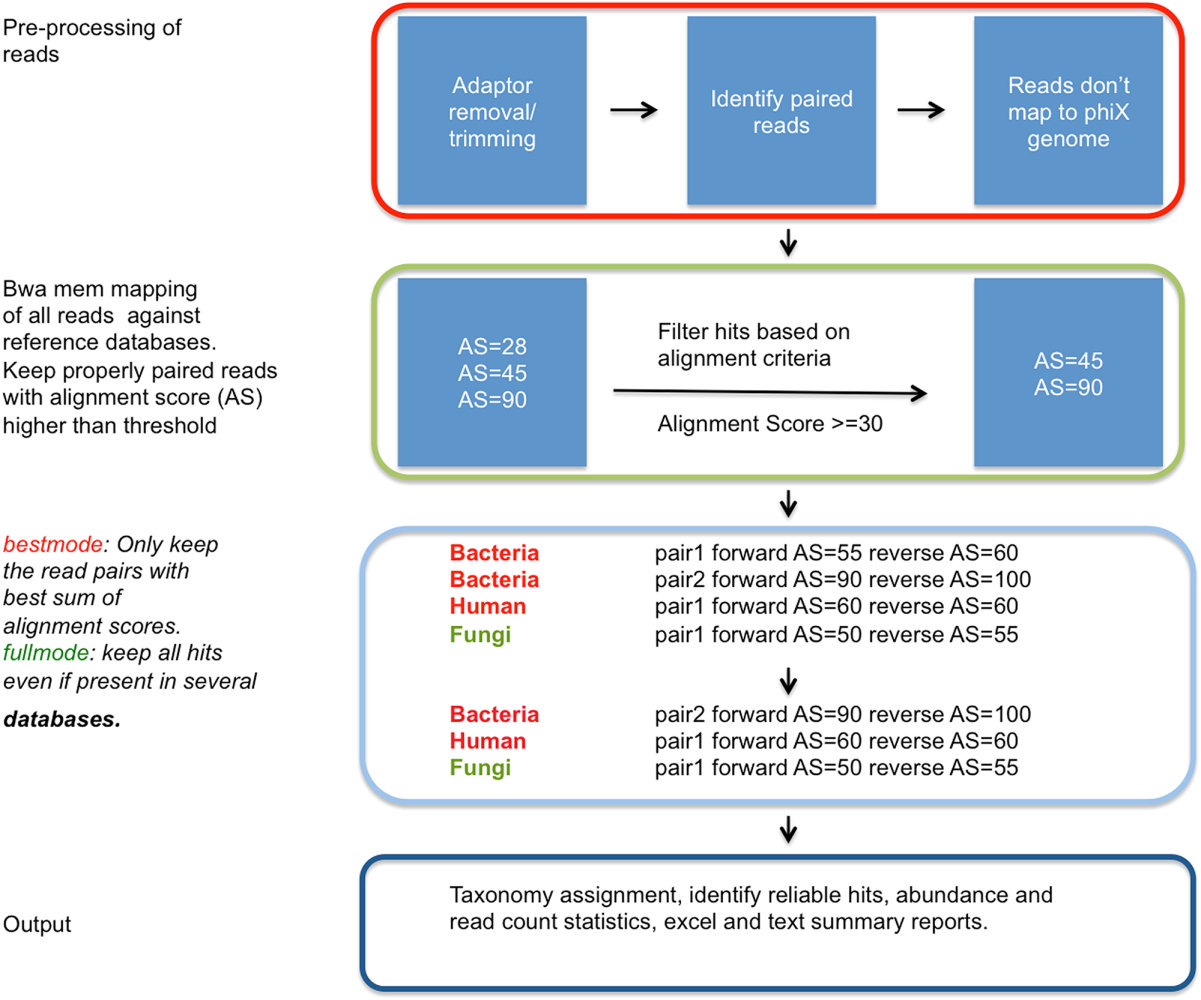
A schematic flowchart for processing of paired-end sequences with MGmapper. MGmapper processes fastq reads in four steps. These consist of: (I) Trimming and mapping reads against a phiX bacteriophage to remove potential positive control reads. (II) Mapping to specified reference databases, post-processing of BWA-mem alignments to remove reads with low alignment score or insufficient alignment coverage. (III) Identification of best hits in *bestmode*: Assignment of a read-pairs to only one specific reference sequence based on the highest sum of alignment scores. In *fullmode*, assigned a read-pair to a reference sequence even if a higher alignment score is found when mapping to another reference sequence database. This will provide best target match, considering only the sequences present in one particular reference database. (IV) Compilation of abundance statistics, read and nucleotide counts, depth, coverage, and summary reports.

The MGmapper pipeline analysis is done in four main steps: I. Pre-processing of raw reads to remove potential positive control reads, II. Mapping of reads to one or more reference sequence databases and filtration of alignment hits, III. Identification the best hits, and IV. Post-processing of taxonomy annotations and preparation of excel and text files with insert-size distribution, size normalized abundances, read and nucleotide count statistics, depth, and coverage. A schematic flowchart of the paired-end mapping processing is shown in Fig 1.

#### I. Pre-processing of raw reads

An optional trimming and filtering of raw reads is performed by use of the Cutadapt [17] program. Users can skip this step if reads are already trimmed. Default setting is that reads are initially trimmed before searching for adaptor sequences (equivalent to the Cutadapt option—q). In addition, a read is discarded unless a minimum of 30 nucleotides remains after trimming. Trimmed reads are next paired up and singleton reads are removed when using the paired-end version of MGmapper. To this follows another cleaning process where reads from potential PhiX Control v3 adapter-ligated libraries are removed via BWA-mem [15] and SAMtools [18], as they may originate from a control for Illumina sequencing runs (http://www.illumina.com/products/phix_control_v3.html). The outcome is a cleaned set of reads that are believed to originate from the biological sample of interest. The number of reads in this set (noPhiX dataset) is set to 100% and used for calculation of R_abundance, a read count abundance measure.

#### II. Mapping of reads to reference sequence databases and alignment based filtering

FASTQ reads are first extracted from the noPhiX set and mapped to one or several reference databases via ‘*bwa mem—t procs—M database’* marking shorter splits as secondary hits, which are then removed when piping to ‘*samtools view -F 256 -Sb -f2’* in paired-end mode or ‘*samtools view -F 260 –Sb’* in single-end mode i.e. keeping properly paired reads or mapped reads, respectively. Next, reads with insufficient alignment qualities are removed based on user-defined minimum alignment score (MAS) and minimum fraction of nucleotides being assigned with an ‘M’ state in the CIGAR format, where an ‘M’ indicates a match or mismatch. The user-defined threshold for fraction of matches+mismatches (FMM) is in relation to the full length of a read. In paired-end mapping both reads are removed if just one of them does not fulfill the filtering criteria. Default settings in the MGmapper programs are MAS = 30 and FMM = 0.8. At this step properly paired read may align to more than one reference sequences, located in different reference sequence databases. In *bestmode* a read pair can only be assigned to one reference sequence (section “*Identification of the best hit*”).

#### III. Identification of the best hit

Having paired-end sequences, both the forward and the reverse fastq reads are aligned to a reference sequence, each with an associated alignment score. The sum of alignment scores (SAS) is used as a measure to identify the best hit for a read-pair. Typically, all input query reads are mapped to multiple reference sequence databases e.g. bacteria, virus, fungi, human and others. Thus a read-pair may map to multiple reference sequences from different databases and in *bestmode* the taxonomy annotation is only assigned to one best hit, namely the one with the highest SAS score.

For single-end reads mapped to several databases, the best hit is the one with the highest alignment score. In cases where a read or read-pair achieves identical alignment scores to reference sequences from different databases, the priority is given to the order by which the databases are specified by the user, and thus a read or read-pair can still be associated to one single reference sequence.

#### IV. Output and post-processing of results

The fastq reads are mapped to multiple user-defined reference sequence databases. A tab-separated file is produced for each database including reference sequence hits with read count statistics provided at strain level. A strain is named according to the header name originating from the fasta file that was used to make the database. The tab-separated file is composed of 14+16 columns of read count statistics and annotations, where the latter are taxid and taxonomy clade name for 8 clades, i.e. strain, species, genus, family, order, class, order and superfamily.

The first 14 columns are described below in Table 2.

**Table 2:** Read count statistics and reference sequence information.

Column identifier	Description
Database	Name of reference sequence database
Ref. seq	Name of reference sequence or clade name
S_Abundance(10^2)	Size normalized read count abundance
R_Abundance(%)	Read count abundance
Size(bp)	Size of reference sequence
Seq_count	Number of sequences in the clade (always 1 at strain level)
Nucleotides	Total number of nucleotides mapped to reference sequence
Covered positions	Number of nucleotide positions covered by the reads
Coverage	Covered positions/size of ref sequence
Depth	Nucleotides/size of ref sequence
ReadCount	Number of reads mapped a ref seq or clade
ReadCount uniq	Number of uniquely mapped reads a ref seq or clade
Mismatches	Number of nucleotide mismatches also known as edit-distance
Description	Description from fasta header or clade name

The size normalized abundance is calculated as S_Abundance = ‘ReadCount x 100/Size’ for single-end reads and ‘ReadCount x100/(2 x Size)’ for paired-end reads. R_Abundance(%) is the number of reads mapped to a taxonomy clade in relation to number of reads after trimming and cleaning versus PhiX genome.

The tab-separated files contains the unprocessed results as obtained by the BWA-mem [15] mapping and Samtools mpileup [18]. As false positive annotations are likely to be present, a subset of confident mapping results is obtained at a specified clade level (strain, species …superfamily) via a post-processing procedure described in the section below.

#### Post-processing

A combination of four criteria (I-IV) is used to identify a positive taxonomy annotation. Identifiers highlighted in italics are also described in Table 2.

I Minimum *ReadCount* of 10
II Mismatch ratio < 0.01, defined as *Mismatches/Nucloetides*.
III *S_Abundance*, the size normalized abundance > 0.01.
IV Unique read count fraction > 0.5%, defined as *ReadCount uniq/ReadCount*.

At strain level all four criteria are imposed. At species level, the criteria IV is used in a pre-cycle, to identify the lowest S_Abundance for the selected species. The new S_Abundance threshold is used in a second round where criteria IV are omitted. At genus level or higher only criteria I, II and III are used.

Taxid values are used to identify strains belonging to the same species or species belonging to the same genus etc. All identifiers as shown in Table 2, are summed at clade levels higher than strain i.e. the S_Abundance value for a species is the sum of all strain S_Abundance values. It is likewise for R_Abundance, Size, Seq_Count, Nucleotides, Covered positions, Coverage Depth, ReadCount, ReadCount uniq and Mismatches.

## Results

Benchmarking of MGmapper at strain, species and genus level against the *in vitro* and the four *in silico* datasets is shown in Tables 3 and 4. Excel sheets are provided as supplementary information in S1–S10 Files, at strain and species level for both annotations that passes the post-processing criteria and for those that are rejected. Also, plasmids sequence annotations are provided in the supplementary excel sheets.

**Table 3:** Benchmarking of the *in vitro* data mapped against a bacteria reference sequence database.

Clade level	TP	FN	FP
Strain	9	3	3
Species	11	0	0
Genus	8	0	0

The columns TP, FN and FP refer to the true positive, false negative and false positive taxonomy annotations for strain, species and genus, respectively.

**Table 4:** Benchmarking of the *in silico* data mapped against a bacteria reference sequence database.

Clade level	TP	FN	FP	Dataset id
Strain	11	2	0	A
Species	12	0	0	-
Genus	9	0	0	-
Strain	11	2	0	B
Species	12	0	0	-
Genus	9	0	0	-
Strain	12	1	0	C
Species	12	0	0	-
Genus	9	0	0	-
Strain	12	1	0	D
Species	12	0	0	-
Genus	9	0	0	-

The columns TP, FN and FP refer to the true positive, false negative and false positive taxonomy annotations for strain, species and genus, respectively. The column ‘Dataset id’ referees to the four datasets A, B, C and D with read lengths of 100bp, 250bp, 500bp and 1000bp, respectively.

A summary of mapping the *in vitro* and *in silico* datasets are shown in Tables 3 and 4, respectively.

For both the *in vitro* data and the four *in silico* datasets, MGmapper identifies all species correctly with no false positive predictions.

The work by Peabody et al. [8] benchmarked several methods at species level using a read count abundance > 0.1% for the *in vitro* data and the *in silico* dataset (although only for the 250bp dataset).

When benchmarked against the *in vitro* dataset, the methods that correctly identified all species with no false positives were a filtered version of Kraken [9] and CARMA3 [10]. For the *in silico* dataset (250bp), six methods performed with no errors i.e. CLARK, Kraken, Kraken filtered, MEGAN4 BLASTN, MetaCV and RITA.

In Table 3 we showed that MGmapper was able to identify all species and genuses present in the *in vitro* dataset without any false positives. Peabody et al., reported that a filtered version of Kraken the same result. Both MGmapper and the filtered version of Kraken need to reject sequence reads in order to report only reliable annotations. In Tables 5 and 6 MGmapper and Kraken have been benchmarked on the percentage of reads that are assigned to each of the genusus and species present in the *in vitro* dataset. The Kraken data was prepared by filtering the read assignments, using a threshold in kraken-filter (version 0.10.6-unreleased-20160118) of 0.2. Next, setting a fair read count abundance threshold for Kraken is not straightforward. We chose to set it at 1.1% such that all species were correctly identified by the Kraken method with no false positives. Also, at genus level a threshold was set at 2.2% to identify all 8 genuses with no false positives. The filters for MGmapper were those described in section ‘Post-processing’. Using these filters the read count abundances are shown in Tables 5 and 6 at genus and species level, respectively. The comparison shows that there is a high degree of similarity between the reported abundances for the individual species and genuses. Also, it shows that fewer reads are mis-classified by MGmapper where a higher total fraction of reads that have been correctly assigned at genus and species clade levels. A strain level comparison was not possible as taxid numbers that are now obsolete and only provided at species level and above.

**Table 5:** Read count benchmark at genus level.

Genus	Read count abundance MGmapper (%)	Read count abundance Kraken (%)
Pseudomonas	34.82	34.98
Bacillus	11.22	11.21
Burkholderia	8.05	7.05
Micrococcus	7.68	7.74
Escherichia	7.53	2.48
Frankia	7.31	7.37
Streptomyces	7.24	7.29
Rhodobacter	6.91	6.96
Total	90.76	87.21

‘Read count abundance’ is reported as the percentage of reads that were assigned to each of the 8 genuses present in the *in vitro* dataset for the two methods; MGmapper and Kraken. Last row ‘Total’ is the overall percentage sum of reads that were assigned to genuses present in the sample.

**Table 6:** Read count benchmark at species level.

Species	Read count Abundance MGmapper (%)	Read count Abundance Kraken (%)
Pseudomonas aeruginosa	15.93	15.93
Pseudomonas protegens	10.81	10.76
Pseudomonas putida	7.95	7.44
Micrococcus luteus	7.68	7.74
Escherichia coli	7.52	2.37
Frankia sp. CcI3	7.31	7.36
Rhodobacter capsulatus	6.91	6.96
Burkholderia cenocepacia	6.17	4.19
Bacillus amyloliquefaciens	5.75	1.96
Streptomyces coelicolor	5.38	4.40
Bacillus cereus	3.40	1.34
Total	84.82	70.45

‘Read count abundance’ is reported as the percentage of reads that were assigned to each of the 11 species present in the *in vitro* dataset for the two methods; MGmapper and Kraken. Last row ‘Total’ is the overall percentage sum of reads that were assigned to the species present in the sample.

## Discussion

The intention behind the development of the MGmapper pipeline is to simplify the processing of next generation sequences from biological samples, and to enable users an easy access to an NGS analysis without necessarily understanding all the computational details of the process. Also, setting up threshold values to provide reliable taxonomy annotations by reducing the number of false positives.

In its present form MGmapper follows a mapping protocols against reference sequence databases, and provide BAM files, text and Excel summary files. These contain read-count statistics for those reference sequences that passed a post-processing procedure, but also for those annotated reference sequences that did not meet the criteria set up in the post-processing, thus enabling a user to see discarded mapping results and possibly redo the post-processing if other threshold settings are preferred. Thus no time consuming fastq mapping needs to be re-done, just the fast post-processing that finishes in seconds.

One of the challenging issues that arise when short sequence reads are mapped against a set of reference sequences is that a read, or a pair of reads, may map equally well to more than one reference in a sequence database i.e. multiple hits with identical alignment scores. When this happens, the reference sequence assignment reported by BWA-mem [15] is arbitrary, as there is not yet any procedure available to unravel the multiple hit ambiguity. However, the fact that there are a number of reads that can be uniquely assigned to one single reference sequence, is a strong indicator that a reported strain or species is actually the one present in the sample. Benchmarking against an *in vitro* dataset, we obtained a sensitivity and precision at 75% for taxonomy annotations at strain level resolution. At higher clade level annotations we identified all species and genuses with no false positives.

The four *in silico* datasets proved easier to annotate correctly compared to the *in vitro* dataset. Only two false negatives were observed at strain level for the 100bp and 250bp datasets and one false negative for the 500bp and 1000bp datasets. At higher clade level annotations, i.e., species and genus, we obtained 100% correct taxonomy annotation. As the most challenging sample was the *in vitro* dataset, we used that in a benchmark analysis to the well performing Kraken method [9]. Overall the MGmapper method correctly assigned 84.82% of the reads at species level, compared to the Kraken method that assigned 70.45% of the reads correctly. At genus level the percentages were 90.75% and 87.21% for MGmapper and Kraken, respectively.

Benchmarking of metagenomics taxonomy classification methods is a challenging effort as programs produce different output and the benchmarking can be done in terms of correctly annotated reads or collapsed into strain/species annotations, run time and memory usage. We chose to compare to the extensive work by Peabody et al. [8], where methods were benchmarked to correctly identify species taxonomy. One of the main results from that study was that all methods, evaluated on an *in vitro* dataset, vastly over predicted the number of species present in a sample unless a post-processing was performed. In this work we have shown that our mapping results together with the post-processing procedure provide 100% correct taxonomy annotations at species and genus level and even at strain level resolution we have trustworthy annotations with sensitivity and precision at 75% when using a combination of up to four criteria to filter the initial mapping results. However, those datasets have limited complexity and for real metagenomics data we can expect much more diversity compared to the 11 and 12 species that are present in the *in vitro* and *in silico* datasets. Metagenomic samples from soil, human gut, sewage or public sites like metro stations, will likely contain a highly diverse set of organisms and also many closely related strains. Reference based sequence alignment methods allow for nucleotide mis-matches between a query read and a reference sequence and the mis-match threshold can be adjusted to assign sequence reads with a remote identity. For practical purposes, a threshold at 10–15% nucleotide mis-matches may be used. A specific organism assigned to be present in a sample may be a representative sequence for closely related sequences with nucleotide variations (SNPs or INDEls). Also Kmer based methods are challenged by highly diverse metagenomics data as they rely on perfect matches between a query fragment and a database hit.

The mapping procedure is the most time-consuming task when running MGmapper. Small datasets that only needs to be mapped against a bacteria, plasmid, fungi or virus databases will finish within minutes up to an hour, whereas mapping millions of fastq reads against many big reference sequence databases like plant (208gb fasta), vertebrate mammals (316gb fasta), invertebrates (150gb fasta) and nt (125gb fasta) will finish days (3–7) even when run in parallel on 16 processors. The *in vitro* and *in silico* datasets used in this study were both run against a bacteria and a plasmid database using 8 cores. The runtime for each of the datasets was 7 min using Computerome—the Danish National Supercomputer for Life Sciences (https://computerome.dtu.dk/).

In future golden benchmark dataset would be most welcome and initiatives like CAMI (Critical Assessment of Metagenome Interpretation) may be a useful platform for benchmark comparison of metagenomics classification tools.

## Supporting information

S1 File Strain and species from in-vitro dataset that pass post-processing filters. (XLSX)

S2 File Strain and species from in-vitro dataset that do not pass post-processing filters. (XLSX)

S3 File Strain and species from in-silico dataset (100bp) that pass post-processing filters. (XLSX)

S4 File Strain and species from in-silico dataset (100bp) that do not pass post-processing filters. (XLSX)

S5 File Strain and species from in-silico dataset (250bp) that pass post-processing filters. (XLSX)

S6 File Strain and species from in-silico dataset (250bp) that do not pass post-processing filters. (XLSX)

S7 File Strain and species from in-silico dataset (500bp) that pass post-processing filters. (XLSX)

S8 File Strain and species from in-silico dataset (500bp) that do not pass post-processing filters. (XLSX)

S9 File Strain and species from in-silico dataset (1000bp) that pass post-processing filters. (XLSX)

S10 File Strain and species from in-silico dataset (1000bp) that do not pass post-processing filters. (XLSX)
