# Correction: MGmapper: Reference based mapping and taxonomy annotation of metagenomics sequence reads

**DOI:** 10.1371/journal.pone.0179778

**Published:** 2017-06-12

**Authors:** Thomas Nordahl Petersen, Oksana Lukjancenko, Martin Christen Frølund Thomsen, Maria Maddalena Sperotto, Ole Lund, Frank Møller Aarestrup, Thomas Sicheritz-Pontén

The fourth author’s name appears incorrectly in the citation. The correct citation is: Petersen TN, Lukjancenko O, Thomsen MCF, Sperotto MM, Lund O, Møller Aarestrup F, et al. (2017) MGmapper: Reference based mapping and taxonomy annotation of metagenomics sequence reads. PLoS ONE 12(5): e0176469. https://doi.org/10.1371/journal.pone.0176469

There are errors in the last sentence of the fifth paragraph of the Results. The correct sentence is: For the *in silico* dataset (250bp), six methods performed with no errors i.e. CLARK [19], Kraken, Kraken filtered, MEGAN4 BLASTN, MetaCV [20] and RITA.

References 19 and 20 should be:

19. Ounit R, Wanamaker S, Close TJ, Lonardi S. (2015) CLARK: fast and accurate classification of metagenomic and genomic sequences using discriminative k-mers. *BMC Genomics*, 16:236.

20. Liu J, Wang H, Yang H, Zhang Y, Wang J, Zhao F, Qi J. (2013) Composition-based classification of short metagenomic sequences elucidates the landscapes of taxonomic and functional enrichment of microorganisms. *Nucleic Acids Res*., **41**, 1–10.
